# Association of oral frailty and its dimensions with cognitive impairment among older adults in China: a cross-sectional study

**DOI:** 10.1186/s12903-025-07507-9

**Published:** 2026-03-19

**Authors:** Yanru Chen, Lisheng Xu, Fan Liu, Ling Zhang

**Affiliations:** 1 State Key Laboratory of Oral Diseases & National Clinical Research Center for Oral Diseases &, Department of Cariology and Endodontics, West China Hospital of Stomatology, Sichuan University, Chengdu, Sichuan Province 610041 China; 2 State Key Laboratory of Oral Diseases & National Clinical Research Center for Oral Diseases &, Department of Prosthodontics, West China Hospital of Stomatology, Sichuan University, Chengdu, Sichuan Province 610041 China; 3 State Key Laboratory of Oral Diseases & National Clinical Research Center for Oral Diseases &, Department of Nursing, West China Hospital of Stomatology, Sichuan University, Chengdu, Sichuan Province 610041 China

**Keywords:** Cognitive dysfunction, Oral frailty, Aged, Cross-sectional studies

## Abstract

**Background:**

Growing evidence highlights that poor oral health may increase the risk of cognitive decline. However, the association of oral frailty and its dimensions with cognitive function remains under-explored. This study aimed to evaluate the association of oral frailty and its dimensions with cognitive impairment among older adults in China.

**Methods:**

This cross-sectional study used convenience sampling to recruit older adults aged ≥ 60 years who received routine oral examinations at the West China Hospital of Stomatology, Sichuan University, from June 2022 to March 2024. Cognitive function was assessed with the Montreal Cognitive Assessment Scale (MoCA), and the oral frailty status was evaluated using the Oral Frailty Index-8 (OFI-8) scale. Binary logistic regression was performed to analyze the independent association between oral frailty and cognitive impairment, with adjustment for potential confounding variables.

**Results:**

A total of 407 older adults were included, revealing a prevalence rate of 42.0% for cognitive impairment. In the fully adjusted binary logistic regression model, oral frailty was associated with an increased risk of cognitive impairment (OR = 1.89, 95%CI: 1.43–2.50). As to the specific dimensions, chewing difficulty (OR = 2.98, 95%CI: 1.86–4.78), low social participation (OR = 4.69, 95%CI: 1.14–19.23), the number of remaining teeth (OR = 0.91, 95%CI: 0.88–0.95), and the GOHAI score (OR = 0.95, 95%CI: 0.92–0.98) were found independent association with cognitive impairment (*p* < 0.05).

**Conclusion:**

Oral frailty is closely associated with cognitive impairment in older adults. Further longitudinal research is required to investigate the underlying mechanisms.

## Introduction

The global demographic of older population is undergoing substantial expansion, with forecasts suggesting that their numbers will increase to 2.1 billion by 2050 and may escalate to 3.1 billion by 2100 [1]. This trend underscores the urgent need for effective prevention and management strategies to address age-related health challenges, positioning it as an important concern within the field of public health [2].

Cognitive impairment, recognized as a prevalent geriatric syndrome, represents an initial phase of memory loss or other cognitive ability loss in individuals who maintain the ability to perform daily activities independently [3]. The recent meta-analysis results of Salari et al. show that the global prevalence of mild cognitive impairment among people aged 60 and above is as high as 23.7% [1], and over half of individuals progress to dementia within 5 years [4]. This condition not only diminishes self-care abilities and quality of life in older adults, but also exacerbates caregiver burdens and healthcare costs [5]. Therefore, timely identification and effective management of modifiable risk factors for cognitive impairment may serve as critical preventive strategies to delay dementia progression and reduce its overall burden [6].

Recently, the relationship between oral health and cognitive decline has become a key focus in geriatric studies. As a significant factor of nutritional intake and social engagement, oral health serves as both a mediator of overall wellbeing and a potential modifiable risk factor for cognitive impairment. The potential mechanism includes the fact that oral activities have an impact on cognitive function by enhancing both brain activity and cerebral blood flow [7]. Epidemiological findings demonstrate this association: severe periodontal disease, persistent toothache, gum pain and tooth loss respectively increase the risk of dementia in older adults by 22%, 38%, 47% and 63% [8–10], while masticatory dysfunction independently correlates with cognitive impairment [11]. As a transitional phase between oral health and oral function decline, oral frailty was recognized as a progressive and cumulative decline in multidimensional oral functions associated with aging [12], may be also intricately associated with cognitive function. However, the majority of existing research has focused on the relationship between individual oral diseases and cognitive outcomes, whereas a systematic investigation of oral frailty and its dimensions from a functional perspective remains lacking. Therefore, this study aimed to assess the association of oral frailty and its dimensions with cognitive impairment in older adults, thereby providing a foundation that could inform future research on early interventions targeting cognitive decline via oral health pathways.

## Methods

### Study design and participants

This cross-sectional study was conducted in adherence to the Strengthening the Reporting of Observational Studies in Epidemiology (STROBE) Statement guidelines [13]. It was approved by the Ethics Committee of the West China Hospital of Stomatology, Sichuan University, China (WCHSIRB-D-2021-482). From June 2022 to March 2024, participants aged 60 and older who sought care at the outpatient clinic of the West China Hospital of Stomatology, Sichuan University, in China were recruited. Individuals with severe intellectual disabilities, hearing or visual problems, and serious physical dysfunction that might lead to inability to complete the assessment were excluded. All participants took part in this study voluntarily and provided written informed consent. Face-to-face structured questionnaire interviews were conducted by professionally trained investigators.

The sample size was estimated through G*Power software (version 3.1.9.7). Based on previous meta‑analysis data indicating a 23.7% global prevalence of cognitive impairment among older adults [1], a significance level of 0.05, a statistical power of 0.80 and a margin of error of 0.05, the minimum sample size was estimated to be 368. Accounting for an estimated 20% dropout rate during the survey, the total number of participants required is at least 334.

### Instruments

#### General information questionnaire

The general information questionnaire was created by the multidimensional potential associated factors of oral frailty in older adults discussed in previous literature reviews [14, 15]. In this questionnaire, we examined age, gender, educational level, marital status, living arrangement, employment status, average annual income of family (million RMB), body mass index (BMI), daily sleep duration, smoking status, alcohol consumption, visual function, hearing function, number of remaining teeth, stroke, diabetes, hypertension, heart disease, Chronic Obstructive Pulmonary Disease (COPD), number of chronic conditions, and number of medications being taken by the older participants.

#### Oral frailty

Oral frailty was assessed based on the Oral Frailty Index-8 (OFI-8) [16]. The OFI-8 consists of 8 oral health related items: Q1: whether it is harder to eat solid food than it was half a year ago (2 points for Yes); Q2: whether they sometimes choke on tea or soup (2 points for Yes); Q3: whether they have false teeth (2 points for Yes); Q4: whether they have dry mouth symptoms (1 point for Yes); Q5: whether the number social outings has decreased compared with half a year ago (1 point for Yes); Q6: whether they can chew hard food, such as peanuts or pickled radish (1 point for No); Q7: whether they have brushed their teeth at least twice a day (1 point for No); and Q8: whether they see a dentist at least once a year (1 point for No). The aforementioned eight items are categorized into five dimensions: chewing difficulties, swallowing difficulties, use of dentures, low social participation and poor oral health behavior. The total OFI-8 score ranges from 0 to 11 points, which scores of 0–2, 3 and ≥ 4 indicate robust, pre-oral frailty and oral frailty, respectively.

#### Cognitive function

The Montreal Cognitive Assessment (MoCA) [17] was used to measure cognitive function, which is a 30-point instrument evaluating seven cognitive domains through 12 task items. Administered in 10–15 min, higher scores on this scale indicate better cognitive capacity. In the present study, the cut-off score is established at 26, and the total score of the measure was adjusted based on the education years, namely one point was added with 12 years or less of education.

#### Physical frailty

The FRAIL (Fatigue, Resistance, Ambulation, Illness, and Loss of weight) Scale [18] was used to assess physical frailty status among older adults which including fatigue, increased resistance or decreased endurance, decreased freedom of movement, coexistence of multiple illnesses, and weight loss. The scale consists of 5 items with a full score of 5. Scores of 0, 1–2 and ≥ 3 is considered non-physical frailty, pre-physical frail and physical frailty, respectively.

#### Depression status

The 15-item Geriatric Depression Scale (GDS-15) [19] was used to measure self-reported depression status of older adults. Users respond in a “Yes / No” format. Scores range 0 to 15, with scores greater than 5 suggesting depression, and higher scores indicate more severe depressive symptoms.

#### Activities of daily living

The Barthel Index (BI) [20]was used to evaluate the participants’ performance in activities of daily living. The BI scale comprises ten items, encompassing personal hygiene, bathing, feeding, toilet use, stair climbing, dressing, bowel control (defecation), bladder control (voiding), ambulation, and chair/bed transfer. The overall score spans from 0 to 100 points and can be categorized into four levels based on the score: Excellent self-care ability (100 points), mild self-care impairment (61–99 points), moderate self-care impairment (41–60 points), and severe self-care impairment (0–40 points).

#### GOHAI

The General Oral Health Assessment Index (GOHAI) [21] was used to assess oral health-related quality of life (OHRQoL). This 12-item instrument measures three dimensions: physical function, pain and discomfort, and psychosocial impact. Total scores range from 12 to 60, with higher scores indicating better OHRQoL.

### Data collection

Prior to conducting the investigation, the investigators underwent unified training on the content and methodology of the questionnaire to ensure consistency in the study. During the research process, the researcher explained the purpose and scope of the study to the participants. Upon securing their consent to participate, questionnaires were distributed. The researchers assisted older adults in completing the questionnaires, providing clarifications and answering any questions raised. Upon retrieval of the questionnaires, the researcher verified that they were fully completed.

### Statistical analysis

All data were analyzed using IBM SPSS 26.0 statistical software. Participants were categorized into two groups based on cognitive status: healthy control (HC) and cognitive impairment (CI). Normal distributions were evaluated graphically and with the Shapiro-Wilk W test. Continuous variables are expressed as Mean ± Standard Deviation (SD) for normally distributed data, or as median with interquartile range (IQR) for non-normally distributed data. Categorical variables are reported in terms of frequencies and percentages. For intergroup comparisons, chi-square tests were used for categorical variables, independent samples *t* - tests for normally distributed continuous variables, and kruskal-Wallis H tests for non-normally distributed continuous variables.

The association of oral frailty and its dimensions with cognitive impairment was examined using binary logistic regression, in which all potential confounding variables identified from univariate analyses (*p* < 0.1) were adjusted for. Three sequentially adjusted models were developed for this analysis: Model 1 adjusted for sociodemographic variables, including age, gender, educational attainment, employment status and average annual income of family. Model 2 additional adjusted for disease-related variables, including hearing impairment, polypharmacy, depression, loneliness and chronic conditions. Model 3 further adjusted for physical function variables, including physical frailty and ADL. All statistical analyses were two-tailed, and statistical significance was set as *p* < 0.05.

## Results

**Fig. 1:**
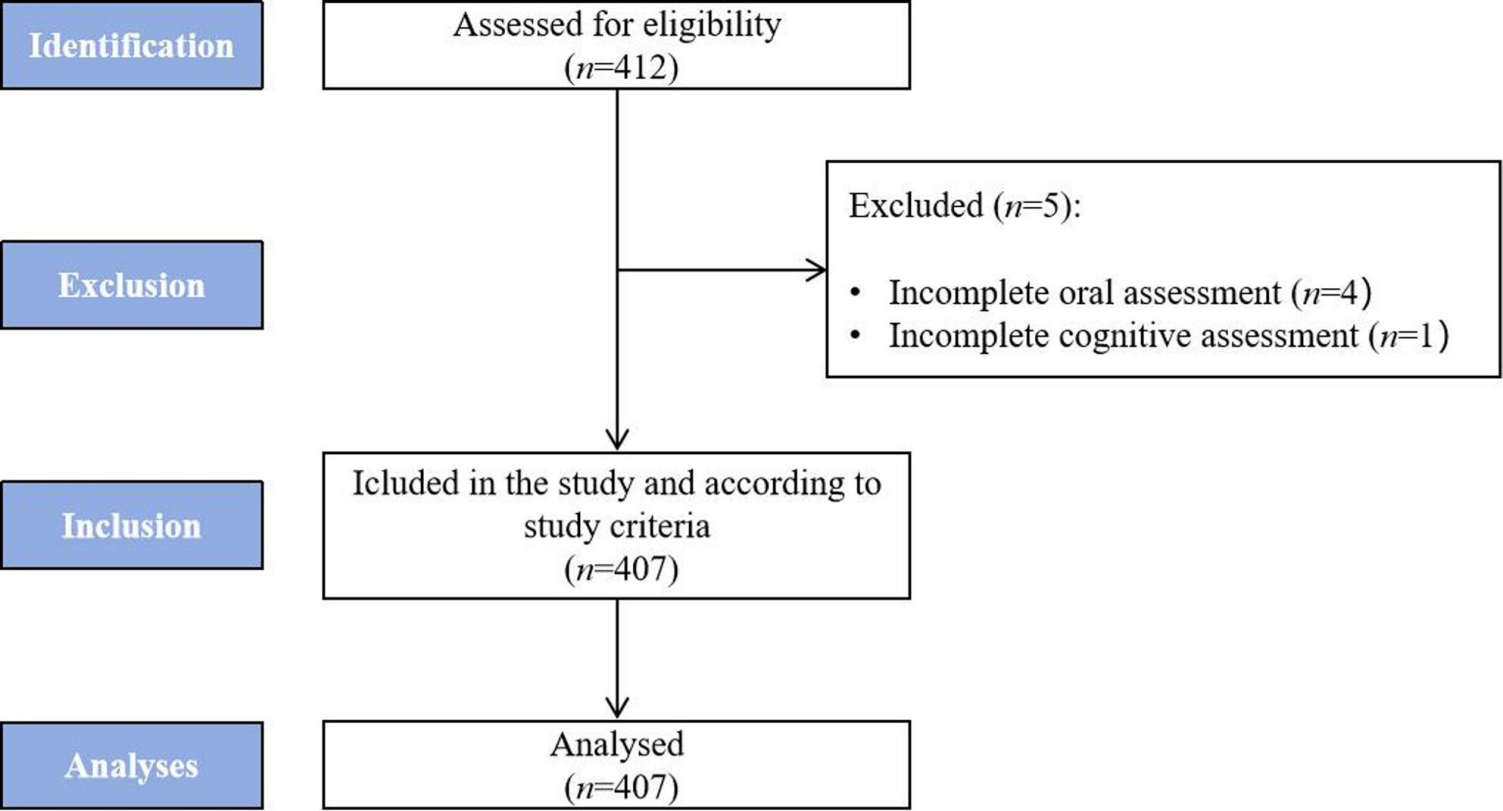
Flowchart of the study participant selection process

A total of 407 older adults who met the eligibility criteria were included and analyzed in this study. A flowchart detailing the participant selection process is presented in Fig. 1.

Comparisons of participant characteristics according to cognitive function are presented in Table 1. The median (IQR) age of participants was 69 (65–75) years, and 57.74% were female. The prevalence of cognitive impairment was 42.0%, and 64.9% of these affected individuals also had oral frailty. Notably, among the dimensions of oral frailty, the most frequently reported issues were chewing difficulties, denture use, and swallowing difficulties. Compared to the healthy control (HC) group, participants with cognitive impairment (CI) were characterized by older age, lower educational attainment, lower annual family income, and a higher prevalence of multiple chronic conditions. Additionally, the CI group exhibited higher unemployment rates and a greater prevalence of loneliness, physical frailty, and ADL impairment. Furthermore, the CI group showed higher OF-8 score and a greater prevalence of oral frailty, which was mainly evident in three dimensions: difficulty in chewing, difficulty in swallowing, and low social participation (all *p* < 0.05).

**Table 1:** Characteristics of the study participants according to cognitive function (*n* = 407)

Variables	Overall (*n* = 407)	HC (*n* = 236)	CI (*n* = 171)	*p*
**Sociodemographic**				
Age, years, Median (IQR)	69 (65–75)	69 (64–74)	70 (65–77)	0.008
Female (%)	235 (57.74)	129 (54.66)	106 (61.99)	0.084
BMI, kg/m^2^, Mean ± SD	23.57 ± 3.15	23.72 ± 3.23	23.37 ± 3.04	0.260
Secondary and below education level (%)	150 (36.86)	66 (27.97)	84 (49.12)	< 0.001
Widowed/unmarried (%)	59 (14.50)	34 (14.41)	25 (14.62)	0.531
Living alone (%)	39(9.58)	24 (10.17)	15 (8.77)	0.384
Unemployed (%)	32(7.86)	9 (3.81)	23 (13.45)	< 0.001
Family average annual income, million RMB, Median (IQR)	2.77 (5.00–7.50)	6.00 (3.50–9.00)	4.00 (2.35–7.20)	0.001
**Health behavior**				
Daily sleep time ≤ 5 h (%)	128 (31.45)	69 (29.24)	59 (34.50)	0.154
Current smoker (%)	43 (10.57)	26 (11.02)	17 (9.94)	0.728
Current alcohol drinker (%)	73 (17.94)	46 (19.49)	27 (15.79)	0.338
**Medical history (presence)**				
Visual impairment (%)	232 (57.99)	130 (55.08)	102 (59.65)	0.207
Hearing impairment (%)	59 (14.50)	29 (12.29)	30 (17.54)	0.09
Stroke (%)	7 (1.72)	4 (1.69)	3 (1.75)	0.625
Diabetes (%)	55 (13.51)	30 (12.71)	25 (14.62)	0.340
Hypertension (%)	130 (31.94)	74 (31.36)	56 (32.75)	0.424
Heart disease (%)	67 (16.46)	36 (15.25)	31 (18.13)	0.261
COPD (%)	10 (2.46)	6 (2.54)	4 (2.34)	0.583
≥ 2 chronic conditions (%)	102 (25.06)	51 (21.61)	51 (29.82)	0.039
Medications ≥ 5 (%)	28 (6.88)	12 (5.08)	16 (9.36)	0.070
Anxiety	119 (29.24)	64 (27.12)	55 (32.16)	0.160
Loneliness	85 (20.89)	36 (15.25)	49 (28.65)	0.001
Depression (%)	28 (6.88)	12 (5.08)	16 (9.36)	0.070
**Physical functional status**				
Pre-PF / PF (%)	110 (27.03)	47 (19.92)	63 (36.84)	< 0.001
ADL (impaired) (%)	87 (21.38)	30 (12.71)	57 (33.33)	< 0.001
**Oral health status**				
Number of remaining teeth, Mean ± SD	21.58 ± 7.01	23.40 ± 5.76	19.06 ± 7.78	< 0.001
GOHAI score, Mean ± SD	48.46 ± 8.02	50.11 ± 7.66	46.19 ± 7.98	< 0.001
Oral frailty				< 0.001
Robust (%)	123 (30.22)	91 (38.56)	32 (18.71)	
Pre-oral frailty (%)	83 (20.39)	55 (23.30)	28 (16.38)	
Oral frailty(%)	201 (49.39)	90 (38.14)	111 (64.91)	
OFI-8 score, Mean ± SD	3.75 ± 2.12	3.28 ± 2.00	4.41 ± 2.11	< 0.001
Oral frailty dimensions				
Chewing difficulties (%)	205 (50.37)	89 (37.71)	116 (67.84)	< 0.001
Swallowing difficulties (%)	155 (38.08)	79 (33.47)	76 (44.44)	0.016
Use of dentures (%)	190 (46.68)	111 (47.03)	79 (46.20)	0.474
Low social participation (%)	17 (4.18)	4 (1.69)	13 (7.60)	0.004
Poor oral health behavior (%)	113 (27.76)	64 (27.12)	49 (28.65)	0.408

*ADL *activities of daily living, *BMI* body mass index, *COPD* chronic obstructive pulmonary disease, *GOHAI* General Oral Health Assessment Index, *IQR* interquartile range, *OF* oral frailty, *OFI-8* Oral Frailty Index-8, *PF* physical frailty, *SD* standard deviation

We constructed three sequentially adjusted binary logistic regression models to evaluate the independent association between oral frailty and cognitive impairment (Table 2). In the fully adjusted model (Model 3), oral frailty (OR = 1.89, 95%CI: 1.43–2.50), chewing difficulties (OR = 2.98, 95%CI: 1.86–4.78), and low social participation (OR = 4.69, 95%CI: 1.14–19.23) were associated with an increased risk of cognitive impairment. Furthermore, fewer remaining teeth (OR = 0.91, 95%CI: 0.88–0.95) and lower GOHAI score (OR = 0.935; 95%CI: 0.909–0.963) were also linked to a higher risk of cognitive impairment in older adults. However, no association was observed between swallowing difficulties and cognitive impairment.

**Table 2:** Association of oral frailty and its dimensions with cognitive impairment in older adults (*n* = 407)

	Model 1			Model 2			Model 3		
	OR	95% CI	*p*	OR	95% CI	*p*	OR	95% CI	*p*
Overall (*n* = 407)									
Oral frailty (yes)	1.94	1.48–2.53	< 0.001	1.90	1.45–2.50	< 0.001	1.89	1.43–2.50	< 0.001
Chewing difficulties (yes)	3.38	2.17–5.28	< 0.001	3.30	2.10–5.18	< 0.001	2.98	1.86–4.78	< 0.001
Swallowing difficulties (yes)	1.48	0.96–2.30	0.078	1.41	0.90–2.22	0.134	1.44	0.90–2.29	0.125
Low social participation (yes)	4.96	1.33–18.57	0.017	4.55	1.17–17.69	0.029	4.69	1.14–19.23	0.032
Number of remaining teeth	0.92	0.89–0.95	< 0.001	0.92	0.88–0.95	< 0.001	0.91	0.88–0.95	< 0.001
GOHAI score	0.94	0.91–0.96	< 0.001	0.94	0.91–0.97	< 0.001	0.95	0.92–0.98	0.001

Model 1: Adjusted for age, gender, educational attainment, employment status and average annual income of family

Model 2: Model 1 plus adjustment for hearing impairment, polypharmacy, depression, loneliness and chronic conditions

Model 3: Model 2 plus adjusted for pre-PF/PF and ADL

## Discussion

In the present study, we investigated the association between oral frailty and cognitive impairment among older adults in China. The results demonstrated an overall association, which is consistent with the findings of Nagatani et al. [22], who reported that oral frailty was associated with a markedly elevated hazard ratio for new-onset mild cognitive impairment compared with other groups, even after adjusting for confounding factors. We further identified that specific dimensions of oral frailty (chewing difficulty, low social participation), as well as the number of remaining teeth and GOHAI score, were associated with cognitive impairment. These findings strengthen existing evidence indicating that accumulated deficits in oral health and functional conditions are associated with cognitive impairment among older adults [23].

Our study revealed that chewing difficulty was the most prevalent dimension of oral frailty, accounting for 50.37% of cases. An alternative conceptualization of oral frailty defines it as difficulties in chewing associated with age-related changes in swallowing [24], which underscores that the preservation or improvement of masticatory function may represent a critical intervention target for the prevention and management of oral frailty. This is particularly relevant as chewing function in older adults is closely linked to the number of remaining teeth, bite force, and salivary flow [25]. In our study, the mean number of remaining teeth among older adults was less than 22, which may be one of the contributing factors to the high prevalence of chewing problems.

Several potential mechanisms may explain the observed association of oral frailty and its dimensions with cognitive impairment among older adults. First, declined oral conditions, such as tooth loss or masticatory dysfunction, may decrease the sensorimotor stimulation produced by the chewing process to the brain [23, 26]. These also provide an explanation for the observed correlation between chewing disorder dimensions and cognitive impairment in older adults. Second, the inflammatory pathway suggests that oral microbiota or inflammatory factors may enter the brain through circulation or neural pathways, increasing the risk of cognitive disorders such as Alzheimer’s disease [27]. Additionally, we found that low social participation was associated with cognitive impairment. Social participation refers to engagement in activities involving interpersonal exchange within the community or society beyond the household [28]. Limited social participation may adversely affect cognitive function by constricting social networks and reducing access to cognitive stimulation, diverse knowledge resources, and emotional support [29].

Furthermore, consistent with previous research, this study found that fewer remaining teeth are associated with an increased risk of cognitive impairment in older adults. This dose-response relationship is corroborated by Kusama et al. [23], who reported that having ≤ 19 remaining teeth and being edentulous were associated with a 1.12-fold and 1.20-fold increased risk of dementia, respectively. These findings substantiate that the number of remaining teeth serves as a significant predictor of cognitive status in older adults [30]. Our results further revealed an association between lower GOHAI scores and cognitive impairment, which is consistent with prior evidence that poor OHRQoL may be an independent risk factor for cognitive decline [31]. The GOHAI scale is used to assess key dimensions of OHRQoL: physical function, pain or discomfort, and psychosocial impact. These dimensions directly reflect oral health deficits, such as tooth loss, dental caries, and functional deterioration [32]. Given that oral health is both readily identifiable and modifiable, these findings underscore the importance of integrating oral health strategies into cognitive preservation efforts in the aging population. Based on the present findings, recommended interventions should include chewing function training, facilitation of social participation, and emphasis on regular dental check-ups and prosthetic rehabilitation to help mitigate cognitive risk.

In summary, our research demonstrates an association between oral frailty and cognitive impairment in older adults. Nevertheless, given that this study employs a cross-sectional design, the determination of causal relationships requires further investigation in subsequent studies, as the relationship may also be bidirectional. Older individuals with cognitive impairment are frequently accompanied by poor oral hygiene awareness and ability, resulting in higher oral bacterial load and inflammation levels, increased risk of dental caries and tooth loss, thus increasing the risk of oral frailty [33]. Future longitudinal studies employing repeated measures are necessary to elucidate temporal precedence. Additionally, intervention studies should be conducted to examine whether specific enhancements in oral health can influence cognitive trajectories in older adults.

This study has several limitations. First, it is a cross-sectional study, which limits any meaningful discussions of causality. Second, the study population was recruited from a stomatology hospital, potentially introducing selection bias. Future research should consider conducting large-scale, multi-center cohort studies to improve the generalizability of the findings. Third, the assessment of oral frailty in this study was based on the OFI-8 scale. Future research should aim to develop more comprehensive assessment tools that also incorporate objective indicators, thereby enhancing the accuracy and comprehensiveness of evaluations. Despite these limitations, this study represents the inaugural investigation into the relationship of oral frailty and its dimensions with cognitive impairment among older adults in China. Elucidating these relationships is essential for informing the prevention of cognitive decline and the comprehensive management of oral frailty among older adults within oral healthcare strategies.

## Conclusions

This study demonstrates an independent association between oral frailty and cognitive impairment in older adults. This finding provides initial evidence and novel perspectives for promoting cognitive health through oral frailty intervention pathways. Further longitudinal studies are necessary to explore the underlying mechanisms.

## Data Availability

The datasets used or analysed during the current study are available from the corresponding author on reasonable request and her email is 22907351@qq.com.

## Abbreviations

ADL: Activities of Daily Living
BI: Barthel Index
GDS-15: 15-item Geriatric Depression Scale
GOHAI: General Oral Health Assessment Index
MoCA: Montreal Cognitive Assessment Scale
OFI-8: Oral Frailty Index-8
OHRQoL: the Oral Health-related Quality of Life
SD: Standard Deviation
